# The Ileoileal intussusception due to a tubular duplication in a child: A case report

**DOI:** 10.1002/ccr3.6759

**Published:** 2022-12-21

**Authors:** Mehran Monazzami, Khashayar Atqiaee, Paria Dehghanian, Hojjat Shaman Zahroodi

**Affiliations:** ^1^ Department of General Surgery, Faculty of Medicine Mashhad University of Medical Sciences Mashhad Iran; ^2^ Department of Pediatric Surgery, Faculty of Medicine Mashhad University of Medical Sciences Mashhad Iran; ^3^ Pediatric pathologist, pathology department Akbar children's hospital Mashhad Iran; ^4^ Student research committee, Mashhad University of Medical Sciences Mashhad Iran; ^5^ Virtual School of Medical Education and Management Shahid Beheshti University of Medical Sciences Tehran Iran

**Keywords:** congenital abnormalities, intestinal obstruction, intussusception

## Abstract

Intussusception is a surgical emergency that may result in the perforation of the intestinal wall if not immediately treated. Pathologic lead points, such as intestinal duplication, are present in 2.2%–15% of the cases.We describe a 4‐year‐old girl with a necrotic ileoileal intussusception diagnosed with a rare tubular ileal duplication.

## Graphical abstract

To ensure the best therapeutic strategy, it is crucial to include small intestinal duplication as a pathological lead point in the differential diagnosis of acute abdomen owing to complicated intussusception.

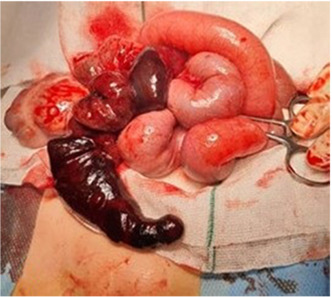

## 1 INTRODUCTION

Intussusception is one of the most common causes of intestinal obstruction. It occurs when a portion of the gastrointestinal tract gets telescoped into another. It can occur in children aged 6 months to 2 years, and the most common type is idiopathic ileocolic intussusception.^1^, ^2^, ^3^, ^4^, ^5^, ^6^ It formerly had significant mortality and morbidity rates, but with advancements in diagnosis and efficient treatment, a good outcome was achieved.^2^ Ischemia and perforation of the intestinal wall are probable in cases where treatment is delayed, with an unfavorable prognosis.^3^

A triad of vomiting, a palpable abdominal mass, and the passage of currant‐jelly stools are the typical signs that raise clinical suspicion of intussusception.^3^ Pathologic lead points such as Meckel's diverticulum, intestinal duplication, benign polyps, malignant lymphoma, Peutz‐Jeghers syndrome, and hamartoma are present in 2.2%–15% of cases.^7^, ^8^, ^9^

The preferred therapy for noncomplicated intussusception is radiological reduction, which has a 10%–60% failure rate and necessitates surgery in failed situations.^3^ Small intestinal duplication is a rare congenital malformation characterized by a tubular or cystic structure that shares its muscular wall and blood supply with the adjacent intestine, primarily the ileum.^10^, ^11^, ^12^, ^13^ This study describes a 4‐year‐old girl with a complicated ileoileal intussusception caused by a tubular ileal duplication.

## 2 CASE PRESENTATION

### 2.1 History

A four‐year‐old Afghan refugee girl was admitted to the pediatric ward with acute abdominal pain as her main complaint and no prior medical or surgical history. The symptoms started as intermittent abdominal pain (every 60 min with a duration of 20 min) in the periumbilical and hypogastric areas 3 days ago, accompanied by two episodes of bilious vomiting (last night and 1 hour before admission) and one episode of currant‐jelly stool defecation with no sign of hematochezia. Her complaints were temporarily relieved by taking anticholinergics and antiemetics as self‐treatment.

### 2.2 Physical examination

In general appearance, she was an ill child, and physical examination revealed an abdominal distention with a palpable tender mass in the periumbilical area.

### 2.3 Laboratory data and imaging study

The laboratory tests revealed leukocytosis (15,600 white blood cells/MCL), neutrophilia (12,948 absolute polymorphonuclear cells/MCL), and a C‐reactive protein of 4.4 mg/dL. Also, urinalysis revealed ketonuria (acetone: 4+, WBC: 4‐6/hpf, epithelial cell: 0‐1/hpf, and a few bacteria).

Ultrasonography confirmed an ileoileal invagination as a doughnut sign with obstructive findings, free abdominopelvic fluid, and no vascular flow in the invaginated loop in Doppler investigations.

### 2.4 Treatment plan

**FIGURE 1:**
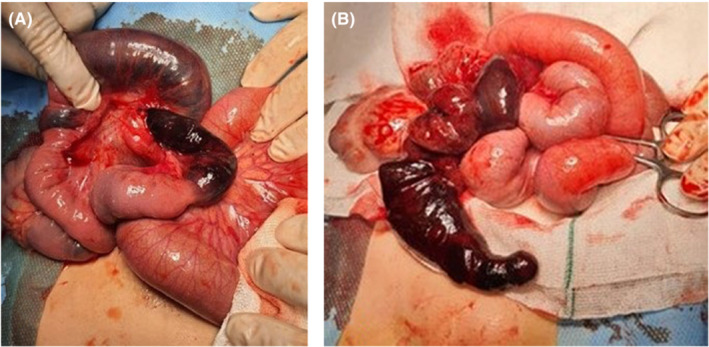
(A) Twisted Invaginated Intestinal Part. (B) The 10 cm necrotic part appeared as the duplication of the Small Intestine

**FIGURE 2:**
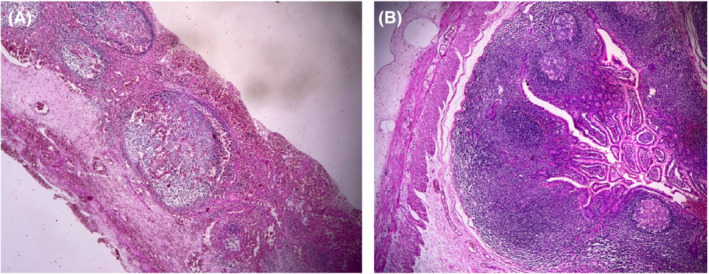
Histopathological findings of the duplicated segment of. (A) Small intestinal wall with hyperplastic lymphoid follicles (H and E staining, low‐power field). (B) Pan‐mural hemorrhagic infarct of the intestinal wall with lymphoid follicles remnants

We performed urgent surgery following fluid resuscitation and the administration of preoperative broad‐spectrum antibiotics due to the patient exhibiting symptoms of peritonitis. The stomach was decompressed using a nasogastric tube while the patient was under general anesthesia. A twisted ileoileal invaginated section was observed at 20 cm from the ileocecal valve during exploratory laparotomy (Figure 1A). First, we attempted to untwist the volvulus, and then, by milking from the distal area, we released the 20 cm invaginated section. Eventually, we found a 10 cm necrotic area as the small intestine duplication (Figure 1B). We decided to perform excision and anastomosis surgery due to the total thickness necrosis and the inability to recover the invaginated part's circulation. The patient was discharged after 96 hours without complications or notable events. Histopathological findings confirmed pan‐necrosis in the resected specimen (Figure 2).

## 3 DISCUSSION

Intussusceptions are typically idiopathic, and only 2.2%–15% have pathologic lead points like Meckel's diverticulum (the most common type) or intestinal duplication.^7^, ^8^, ^9^

Most pathologic lead points present as ileoileocolic intussusceptions, and generally, signs and symptoms are similar; however, due to the large variety of lesions or intestinal malformations, presentations might vary and are frequently nonspecific. Pathologic lead points should be considered, especially in children over 5 years with multiple recurrences or failure of the enema technique for reduction.^3^

We recommended that under suspicious circumstances, a primarily negative ultrasonography investigation should never exclude a second repeat ultrasonography.

Duplications of the small intestine are common in the ileum and can be cystic or tubular. They differ from Meckel's diverticulum in that they are mostly linked to the mesenteric region of the intestine and consist of two parts as follows: an outer layer of smooth muscle and an inner lining of the gastrointestinal epithelium.^2^, ^10^, ^12^, ^13^, ^14^

As many children with intussusceptions caused by pathologic lead points may present with nonclassic symptoms, a specific diagnosis must be determined by diagnostic examination using radiologic imaging. The best options for confirming intussusception caused by a duplication cyst or lymphoma in the hands of an experienced examiner are ultrasonography and reduction enema, but less so for a Meckel's diverticulum.^2^, ^3^, ^4^, ^5^, ^7^, ^8^, ^9^

Treatment of a stable patient with intussusception begins with an enema reduction in the emergency department without contraindications such as perforation or shock signs.^3^

Our case presented with the classic symptoms of intussusception, and the ultrasonography findings boosted the suspicion. We identified the tubular type of ileal duplication at the operation, which caused ileoileal intussusception. In the literature, we found a few cases of children with ileoileal intussusception secondary to tubular ileal duplication.^5^, ^15^

## 4 CONCLUSION

Small intestine duplication should be considered in the differential diagnosis of ileoileocolic or ileoileal intussusception, especially in children over 5 years with failed reduction attempts. Moreover, surgical treatment is imperative for all symptomatic cases.

## AUTHOR CONTRIBUTIONS

**Mehran Monazzami:** Writing – review and editing. **paria Dehghanian:** Writing – review and editing. **Hojjat Shaman Zahroodi:** Writing – original draft.

## CONFLICT OF INTEREST

The authors declare that they have no competing interests in this original work.

## ETHICAL APPROVAL

We confirm that all named authors have read and approved the manuscript. The protection of the intellectual property associated with this manuscript has been our consideration.

## CONSENT

The authors confirmed that they had gotten all proper patient written consent formats. The parents have given their consent for their images and other clinical Information to be reported in the form. The parents were informed about the confidentiality of the names and initials, and efforts would be made to hide their identity.

## Data Availability

Data sharing is not applicable to this article as no new data were created or analyzed in this study.
